# Local Defense Factors in Cleft-Affected Palate in Children before and during Milk Dentition Age: A Pilot Study

**DOI:** 10.3390/jpm14010027

**Published:** 2023-12-25

**Authors:** Laura Ozola, Mara Pilmane

**Affiliations:** Institute of Anatomy and Anthropology, Riga Stradins University, Kronvalda Boulevard 9, LV-1010 Riga, Latvia

**Keywords:** cleft palate, tissue defense factors, milk dentition, HBD, LL-37, IL-10, CD-163

## Abstract

One of the most frequent congenital orofacial defects is the cleft lip and palate. Local tissue defense factors are known to be important in immune response and inflammatory and healing processes in the cleft tissue; however, they have only been researched in older children during mixed dentition. Thus, the aim of this study is to assess the distribution of LL-37, CD-163, IL-10, HBD-2, HBD-3, and HBD-4 in children before and during milk dentition. The unique and rare material of palate tissue was obtained from 13 patients during veloplastic surgeries during the time span of 20 years. Immunohistochemistry, light microscopy, semi-quantitative evaluation, and non-parametric statistical analysis were used. A significant decrease in HBD-3 and HBD-4 in the connective tissue was found, as well as several mutual statistically significant and strong correlations between HBD-2, HBD-3, HBD-4, and LL-37. Deficiency of HBD-3 and HBD-4 suggests promotion of chronic inflammation. The scarcity of HBD-4 could be connected to the different signaling pathways of dental pulp cells. Mutual correlations imply changes in the epithelial barrier, amplified healing efficiency, and increased antibacterial line of defense. Deprivation of changes in IL-10 quantity points to possible suppression of the factor. The presence of similar CD-163 immunoreactive substances produced by M2 macrophages was also observed.

## 1. Introduction

Orofacial clefts (OFC) are frequently occurring multifactorial birth defects that are defined by the presence of aberrant gaps that can develop in the upper lip (CL), palate (CPO), or upper lip and palate simultaneously (CLP) [1,2]. Based on the characteristics of the affected cleft area, they are classified as either bilateral or unilateral and complete or incomplete [1,2].

Orofacial clefts are one of the most prevalent inborn abnormalities in newborns across the world, with an average occurrence rate of 1 per 700 newborns [1,2,3,4]. The incidence of clefts varies based on race and ethnicity. CLP is more prevalent in Asian and European populations and less prevalent in African populations; however, CPO occurs equally throughout various ethnicities and populations [1,5]. In Europe, OFC incidence is more frequent in Scandinavian and Northern European countries compared to other parts of Europe [6,7]. Moreover, it has been observed that the incidence of CLP is more frequent in males, but CPO occurrence frequency is much more prominent in females [1,5,7].

OFCs are mainly formed in the presence of any type of disruptions in facial tissue cell migration, apoptosis, and differentiation or in the mechanisms of fusion, movement, and merging of the facial processes that occur during the developmental process of the orofacial region in the time period from fourth to twelfth week of embryological development [1,2,4,8]. A cleft lip is mostly caused by an incomplete fusion of maxillary and medial nasal processes, while a cleft palate is caused by a disrupted fusion of the palatal shelves [4,8,9].

Most commonly, these developmental disruptions are caused by various genetic factors that are reinforced and amplified with several teratogenic factors occurring during pregnancy, such as malnutrition, use of alcohol or tobacco, abnormal hormone levels, and consumption of various toxins and medications [4,8,10].

Due to the nature of the defect, proper feeding, nutrition, breathing, and orofacial region development is impaired for the newborn [1,10]. Additionally, the equilibrium of bacterial microbiota changes, and, therefore, the orofacial region becomes more susceptible to various bacteria-induced diseases and medical complications, such as lower respiratory tract infections, caries, gingivitis, and opportunistic pathogen infections [11]. Therefore, surgical treatments like cheiloplasty and/or palatoplasty are necessary to restore the functions and aesthetics of the orofacial region, as well as various post-surgery treatments, such as speech therapy, orthodontic correction of the occlusion and even follow-up surgery in case of post-operation complications are often required [2,5].

It is also known that in children with orofacial clefts, the prevalence of inflammatory processes in the orofacial zones is much more prominent; hence, tissue remodelation and healing of the clefted areas is dysfunctional due to the constant presence of chronic inflammation [12]. This raises the question of how and what type of cytokines and antimicrobial peptides and their deviations from the natural equilibrium promote the atypical and prolonged immune response of the clefted tissue.

LL-37 cathelicidine is released in the epithelial tissue, bones, and bone marrow [13]. Its release is mainly carried out by keratinocytes, neutrophils, epitheliocytes, monocytes, macrophages, leukocytes, and B-lymphocytes, as well as gingival, salivary gland, respiratory, gastrointestinal tract, and urinary pathways forming and coating epithelium [13,14]. Cathelicidine functions as a part of innate immunity, implementing structures that destroy the membranes of the pathogen microorganisms that have infiltrated the body by forming pores in membranes and, therefore, destroying these organisms [13,15]. It effectively functions against bacteria, viruses, fungi, and parasites. Moreover, a high concentration of cathelicidine in the body can induce the destruction of already preexisting biofilms and the prevention of new biofilm formation [16,17]. In addition, it also ensures the implementation and control of organisms’ inflammatory response with increased chemotaxis, cell proliferation, phagocytosis, regulated cell differentiation, inflammatory cytokine secretion, various cell apoptosis, and parallelly induced tissue healing and angiogenesis [13,15]. In the realization of tissue healing and angiogenesis processes, the cooperation of LL-37 and human beta-defensin 3 (HBD-3) is also important because both molecules promote epithelial cell and keratinocyte migration, differentiation, and proliferation [18]. In the effect of LL-37, there is an increase in mast cell, neutrophil, monocyte, and lymphocyte infiltration and degranulation in tissues, and also in IL-12 and IL-8 secretion, but a decrease in interleukin 10 (IL-10) secretion [15,19]. Therefore, it promotes inflammation reaction and functions as a proinflammatory mediator [15,19]. In the oral cavity, LL-37 is significant in the maintenance of bacterial equilibrium homeostasis and the prevention of dysbacteriosis.

M2 CD-163+ is a type of macrophage whose main role is to carry out tissue regeneration and control the immune response [20,21]. Macrophages form conglomerates in tissue that has been affected by an injury and/or an infection. There, they function as antigen-identifying and phagocytosing cells, as agents that mediate revascularization, tissue regeneration, and inflammatory processes in the body and also carry out the immune system’s transition from innate to adaptive immune response [20,21]. The M2 phenotype of macrophages is mainly linked to anti-inflammatory actions and Th2-related immune response [20,21]. M2 is crucial in the healing processes and in the secretion of IL-10, arginase I, and TGF-β, which promote angiogenesis and the termination of inflammatory responses [21,22,23]. Immunohistochemically, M2 macrophages are identified by their specific membrane CD-163 hemoglobin scavenger receptors [20]. Increased CD-163 amounts in the tissue are linked to M2 macrophage activity [20]. CD-163 expression is accelerated by M2 macrophage-secreted anti-inflammatory cytokines [24].

IL-10 reduces inflammation in the case of tissue damage and also enhances the action of innate immunity and tissue regeneration mechanisms [25]. The main function of this cytokine, therefore, is to ensure immunosuppression and tissue homeostasis, which has been disrupted by the inflammatory process’s mediated tissue damage [25]. IL-10 also ensures that the inflammatory reaction against pathogens that have entered the body is not extensively overactive and does not cause significant and non-reversible tissue damage [26]. The main IL-10-secreting cells are leukocytes, which, depending on the type of immunity or stage of inflammatory reaction, can be differentiated either as T or B lymphocytes, NK cells, macrophages, or neutrophils [25]. Therefore, IL-10 can be associated with an inflammation-inhibiting action and increased CD8+ cytotoxic activity in the tissue, which results in a noticeable anti-tumor directed action and activity [25,27]. Because the main role of IL-10 is to stop inflammation, interferences and disturbances in its activation pathways often result in prolonged and chronic inflammatory diseases [25].

Human beta-defensin 2 (HBD-2), HBD-3, and human beta-defensin 4 (HBD-4) are the main defensins secreted by the oral cavity and gingival epithelium [28]. They are also found in saliva, and gingival crevicular fluid, and the secretion of these defensins is initiated only by infection or inflammation [28].

HBD-2 is an antimicrobial peptide whose main role is to shape innate immunity in the case of inflammation and to promote the healing of wounded tissue [29]. It participates in the immune response by boosting phagocytosis, proliferation, and the cells of the immune system, as well as by decreasing the secretion of macrophage-made proinflammatory cytokines and by decreasing the activity of C’ complement system in order to impede its unreasonable and inadequate activation during the realization of innate immunity and to promote the chemotaxis of various cells [28,30,31]. The spectrum of its functions also includes the reconstruction of the mechanical barrier of the skin [28].

HBD-3 has similar properties in carrying out innate and adaptive immunity to HBD-2 [32]. The specific characteristic property of HBD-3 in the realization of the immune response is their ability to induce NK cell activity through TLR1 and TLR2 receptors that are expressed in these cells [32]. While the immune response takes place, HBD-3 also induces cells like keratinocytes to migrate and proliferate [28]. Furthermore, it can influence fibroblast activity and the release of angiogenetic growth factors, resulting in faster healing of tissue and wounds [28].

HBD-4 has an inherent ability to bind to lipopolysaccharide structures of bacteria cell walls; therefore, in the process of inflammation, it provides high antimicrobial activity [33,34]. When binding to LPS, HBD-4 decreases proinflammatory actions induced by pathogenic microbes, for example, by lowering IL-6, IL-1α, TNF- α, and TLR2 expression [34].

Because HBD-2, HBD3, and HBD-4 are crucial components of the inflammation-induced immune response of the body, and the increase in their levels is distinctly observed in the case of acute inflammation, these peptides can be potentially used as a diagnostic tool and a therapeutic method that helps the epithelium of the oral cavity and the gingiva to heal faster and more effectively after an inflammation or surgical operations and manipulations [31].

Although changes in tissue defense factor levels in clefted tissue have been researched before, the data are scarce. In children ages 6 to 12, there is a statistically significant increase in HBD-2, IL-10, and HBD-4 levels and a mutual correlation between human beta-defensins, cathelicidine, and interleukin 10 [35]. This data suggests that in the case of clefted tissue, human beta-defensins regulate the inflammatory process and promote healing, but IL-10 suppresses the overly active inflammation of the tissue [35].

However, the presence of local tissue defense factors and their connection to the genesis of orofacial clefts and its associated inflammatory process has only been studied in populations with permanent or mixed dentition. Therefore, in order to expand the knowledge about possible causalities between cleft morphopathogenesis and tissue defense factors and determine what type of changes in defense factor quantities are present in clefted tissue, the present study aims to examine the distribution of local tissue defense factors in cleft lip and palate tissue for children before (birth–6 months) and during milk dentition (6 months–3.5 years).

## 2. Materials and Methods

### 2.1. Material Characteristics of Subjects

This research was conducted in accordance with the 1975 Helsinki Declaration (as revised in 2008). The study was independently reviewed and approved by the Ethical Committee of the Riga Stradiņš University (22 May 2003; 17 January 2013; 5/25 June 2018). Written informed consent for participation in the study and publication of the study data was obtained from all the patients, which was given by parents after the nature of the study had been fully explained.

In total, 13 patient tissue samples of soft palate were obtained during veloplastic surgery in the Cleft Lip and Palate Centre of the Institute of Stomatology of Riga Stradins University from children aged 8 to 12 months diagnosed with unilateral or bilateral cleft lip and palate (Cheilognathouranoschisis dextra/sinistra/bilateralis).

#### 2.1.1. Characteristics of Tissue Samples

The total area of each obtained tissue sample was approximately 2 mm^2^. The relatively small size of the acquired samples represents the amount of tissue material that was not needed for the patient during veloplastic surgery and was extra. If the surgery requested all available tissue material to be used to close the clefted defect successfully, no samples were taken.

#### 2.1.2. Characteristics of Selected Patients

Overall, 6 of these patients were male, and 7 were female; the average age of the cleft-affected patients was 9 months. The CLP of the right side was diagnosed for 5 of these children, CLP of the left side—for 5 children, and CLP of both sides—for 3 children. Also, 2 of the patients had mothers with unfavorable influences during pregnancy—the use of paracetamol or smoking during pregnancy. One patient’s mother was also diagnosed with CLP. All patients underwent veloplastic surgery (Table 1).

### 2.2. Selection Criteria of Patient Tissue Samples

Samples were selected using the inclusion criteria of (1) diagnosis of Cheilognathouranoschisis dextra/sinistra/bilateralis, (2) age before or during milk dentition [36], (3) both genders, (4) absence of other congenital diseases, (5) absence of additional pathology that would contraindicate veloplastic surgery, (6) no visible inflammation, (7) indication for plastic surgery and the exclusion criteria of (1) age during mixed or permanent dentition, (2) presence of multiple congenital diseases, (3) presence of additional pathologies that contraindicate plastic surgery, (4) visible signs of inflammation.

The inclusion criteria of patient age before or during milk dentition was selected because this age is crucial to the development of the oral cavity. Developmental processes during this age are highly affected by the eruption of milk teeth; therefore, significant differences and variations can be observed when compared to the age of mixed dentition. Moreover, this study broadens the field of research connecting CLP and local tissue defense factors, whereas previous studies have only been conducted in older children during mixed dentition [35]. No major limitations were related to the choice of patient age because both age groups (before and during milk dentition and during mixed dentition) have been chosen as subjects of research. The only minor potential limitation could be that acquisition of the unique tissue material during veloplastic surgery is highly challenging due to the fact that almost all of the material available is necessary for the surgeon to repair the patient’s orofacial defect successfully. The young age of the patient also significantly contributes to the scarcity of tissue material. Therefore, even with the lengthy tissue acquisition process throughout the time period of 20 years, the availability of excess and unessential tissue material may be scarce, and the obtainment of only 13 samples was possible. This extreme difficulty in obtaining the tissue samples and the specific age group from before and during milk dentition points out the significance and uniqueness of the material used in this study.

### 2.3. Selection Criteria of Control Tissue Samples

The control group tissue samples were collected from the Institute of Anatomy and Anthropology of Riga Stradins University and consisted of post-mortem necropsies. The 5 control tissue samples of soft palate were obtained from 2 newborns that were affected by sudden death syndrome, 2 newborns that experienced asphyxia by the umbilical cord, and one 24-week-old individual that had been aborted due to the status of the maternal health (Table 2). The inclusion criteria for the control group were the following: (1) the absence of craniofacial clefts found on patient examination or in the patient’s anamnesis and family history and (2) the absence of any other pathologies, congenital abnormalities, or oral cavity tissue damage upon the clinical investigation of the body. The approval Nr. 2-PEK-4/492/2022 for the use of control group tissue was issued on 21 November 2022. The approval Nr. 2-PEK-4/595/2022 for the use of control group tissue was issued on 14 December 2022.

All of the examined patient and control group tissue samples were provided as a donation with the authorization and permission of the deceased and the orofacial cleft patient’s parents. Due to the fact that the control and patient group tissue samples belonged to children in the age group before milk dentition, both groups were considered to be mutually comparable.

### 2.4. Routine Staining

In order to fixate the obtained tissue samples, a solution of 2% formaldehyde, 0.2% picric acid, and 0.1 M phosphate buffer with a pH value of 7.2 was used. Subsequently, a 12 h long washing procedure with phosphate buffer, saline solution, and 10% saccharose was performed. Lastly, the samples of tissue material were embedded in paraffin and sectioned into 5–7 μm thin sections. Hematoxylin and eosin staining were performed to assess the routine morphology of the samples [37].

### 2.5. Immunohistochemical (IHC) Analysis

Standard streptavidin and biotin immunostaining methods [35,37] were used to prepare the tissue samples and immunohistochemically detect the quantity of LL-37, CD-163, IL-10, HBD-2, HBD-3, and HBD-4 tissue defense factors present in the selected tissue specimens. In order to achieve antibody dilution, an antibody diluent (code-938B-05, Cell MarqueTM, Rocklin, CA, USA) was used. Washing with TRIS buffer, blockage with 3% peroxide solution, and repeated washing with TRIS buffer was performed. Subsequently, 1 h long incubation with primary antibodies and washing with TRIS buffer three times followed. After the use of the HiDef Detection™ reaction amplificator (code 954D-31, Cell MarqueTM, Rocklin, CA, USA) for 10 min, the tissues were again washed with TRIS buffer. Following incubation with HiDef DetectionTM HRP Polymer Detector (code-954D-32, Cell MarqueTM, Rocklin, CA, USA) for 10 min and then washed with TRIS buffer 3 times afterward. The tissue samples were then coated with DAB+ chromogenic liquid DAB Substrate Kit (code 957D-60, Cell MarqueTM, Rocklin, CA, USA) for 10 min, rinsed under running water, and counterstained with hematoxylin (code-05-M06002, Mayer’s Hematoxylin, Bio Optica Milano S.p.A., Milano, Italy). As the last step of the immunohistochemical staining, dehydration with increasing concentrations from 70° to 90° of ethanol was performed, with the following steps of clarification with carboxylic acid and xylol and sealing with a coverslip.

#### 2.5.1. LL-37

Detection of LL-37 (orb88370, working dilution 1:100, Biorbyt LLC, St Louis, MO, USA) was performed [35]. This defense factor is important in the pathogenesis of CLP because it is one of the main immune system elements that protects the mucosa by directly destroying pathogens and their membranes [13,14,15].

#### 2.5.2. CD-163

Detection of CD-163 (ab87099, working dilutions 1:200, Abcam, Cambridge, UK) was performed [35]. CD-163 is an important antigen that marks antimicrobial M2 macrophages, whose main role is to perform tissue healing and immune response functions, especially in tissues where inflammation is present [20,21].

#### 2.5.3. IL-10

Detection of IL-10 (orb100193, working dilution 1:600, Biorbyt LLC, St Louis, MO, USA) was performed [35]. IL-10 is one of the most powerful anti-inflammatory cytokines that renews tissue homeostasis of inflammatory and damaged tissue by implementing immunosuppressive actions [25].

#### 2.5.4. HBD-2

Detection of HBD-2 (sc-20798, working dilution 1:100, Santa Cruz Biotechnology Inc., Dallas, TX, USA) was performed [35]. HBD-2 is a universal defensin that is secreted by the epithelial cells of the oral cavity and gingiva [28]. Its main function is to regulate and balance the immune responses of the tissue [29].

#### 2.5.5. HBD-3

Detection of HBD-3 (orb183268, working dilution 1:100, Biorbyt LLC, St Louis, MO, Children 2023, 10, 1162 4 of 15 USA) was performed [35]. This defense factor acts similarly to HBD-2, but its course of action is more connected to increased tissue healing and the elimination of specific types of bacteria [28,32].

#### 2.5.6. HBD-4

Detection of HBD-4 (ab70215, working dilution 1:100, Abcam, Cambridge, UK) was performed [35]. HBD-4 is an essential defense factor that ensures protection of the tissue by increased antimicrobial effects, stimulated IL-10 secretion, and improved tissue healing cell proliferation [33,34,38,39].

### 2.6. Assessment of Local Tissue Defense Factor Quantity

The semi-quantitative counting method and light microscopy method were applied to assess the relative quantity of LL-37, CD-163, IL-10, HBD-2, HBD-3, and HBD-4 positive structures in the epithelium and connective tissue of the prepared tissue sample slides [40,41]. Positively stained structures visible in the visual field were evaluated by the identifiers summarized in Table 3. Illustrative pictures of the stained tissue samples were taken with a Leica DC 300F digital camera (Leica Microsystems Digital Imaging, Cambridge, UK) and processed and analyzed with the Image Pro Plus program (Media Cybernetics, Inc., Rockville, MD, USA).

### 2.7. Statistical Analysis

Statistical data processing was performed using the IBM SPSS (Statistical Package for the Social Sciences) software version 26.0 (IBM Company, Chicago, IL, USA). For every statistical assessment of the tests and results, a *p*-value < 0.05 was selected as statistically significant [42]. Statistical analysis of the data was performed using methods of descriptive and analytical statistics. Since the tissue defense factor levels were evaluated using the semi-quantitative method, the obtained data were ordinal (non-numeric and arranged in a specific and unchangeable order). Therefore, non-parametric tests were used to calculate the results.

#### 2.7.1. Mann–Whitney U Test

The Mann–Whitney U test was performed to detect statistically significant differences in defense factor levels in the patient group and control group samples. The test shows whether the distribution of variables in 2 separate groups is equal or not. Therefore, by using it, it was possible to assess if the mean quantity of observed positive structures in the patient group and in the control group were statistically significantly different [42].

#### 2.7.2. Spearman’s Rank Correlation

Spearman’s rank correlation was used to determine and evaluate statistically significant correlations between human beta-defensins and cathelicidine. The test shows the rate at which the changes in one factor are connected to the changes in other factors [42].

To assess the strength of the correlation, the following definition of Spearman’s rho (r_s_) values was used, where r_s_ = 0.00–0.19 was interpreted as a very weak correlation, r_s_ = 0.20–0.39 as a weak correlation, r_s_ = 0.40–0.59 as a moderate correlation, r_s_ = 0.60–0.79 as a strong correlation, and r_s_ = 0.80–1.00 was interpreted as a very strong correlation [4].

**Figure 1:**
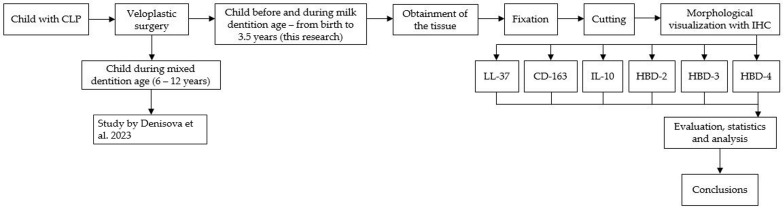
Flowchart illustrating the sample selection and processing steps [35].

The summary of all the steps taken that have been described in the section on materials and methods are outlined in Figure 1.

## 3. Results

### 3.1. Routine Staining

**Figure 2:**
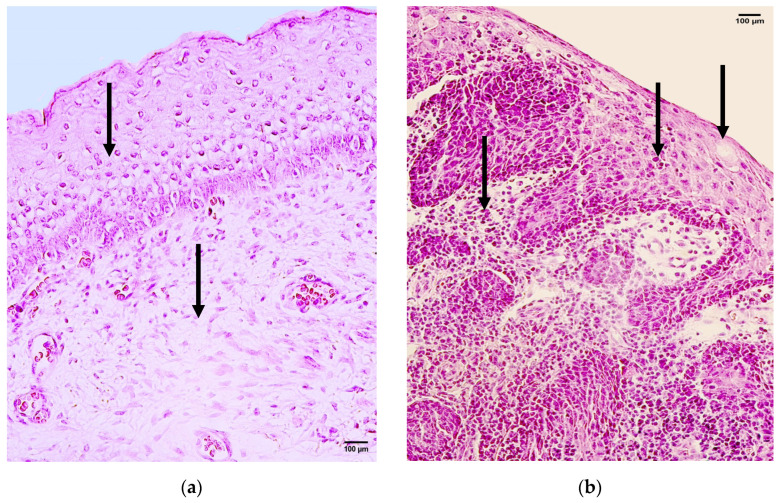
Hematoxylin and eosin routine staining of the control and patient tissue samples. (**a**) Control sample with unchanged non-keratinized stratified squamous epithelium and underlying mucosal connective tissue (arrows). Hematoxylin and eosin, 200×; (**b**) patient sample with numerous intra-epithelial and subepithelial inflammatory cell infiltration and rare occurrence of epithelial vacuolization (arrows). Hematoxylin and eosin, 200×.

The control showed no deviance from the features of standard healthy tissue—the presence of normal non-keratinized stratified squamous epithelium and underlying mucosal connective tissue was observed (Figure 2a). The patient group tissue displayed patchy epithelial vacuolization, hyperplasia of basal cells, and subepithelial infiltration of inflammatory cells (Figure 2b).

### 3.2. LL-37

**Figure 3:**
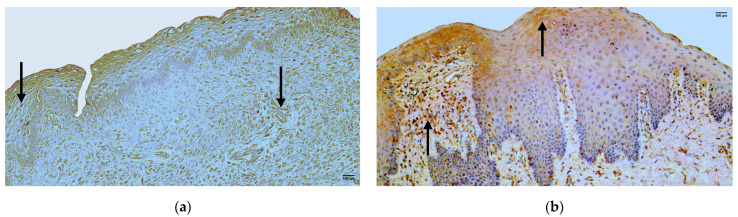
Immunohistochemistry of the LL-37 positive structures in the control and patient tissue samples. (**a**) Control sample with moderate LL-37 positive structures in the epithelium and moderate LL-37 positive structures in the connective tissue (arrows), LL-37 IMH, 200×; (**b**) patient sample with moderate to numerous LL-37 positive structures in the epithelium and numerous LL-37 positive structures in the connective tissue (arrows), LL-37 IMH, 200×.

In the control group, the median quantity of the LL-37 positive structures in the epithelium was few to moderate (+/++) with fluctuation from none to moderate to numerous, but in the connective tissue—it was moderate (++) with no fluctuations (Figure 3a, Table 4).

The median quantity of LL-37 positive structures in the epithelium of the patient group was few to moderate (+/++), with fluctuations from none to abundant. The median quantity of LL-37 positive structures in the connective tissue of the patient group was moderate (++), with fluctuations from none to numerous (Figure 3b, Table 5).

A comparison of the control group and patient group using the Mann–Whitney U test illustrates no statistically significant difference (U = 27.5, *p* = 0.799) in the structures of the epithelium and no statistically significant difference (U = 25, *p* = 0.391) in the structures of the connective tissue (Table 6).

### 3.3. CD-163

**Figure 4:**
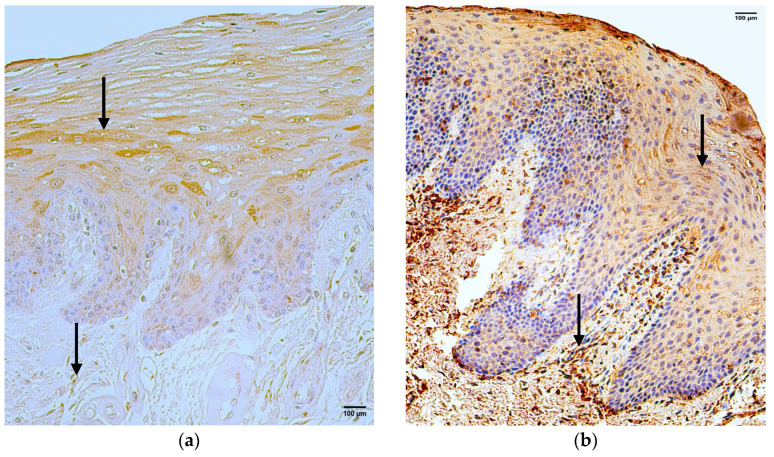
Immunohistochemistry of the CD-163 positive structures in the control and patient tissue samples. (**a**) Control sample with numerous CD-163 positive structures in the epithelium and rare CD-163 positive structures in the connective tissue (arrows), CD-163 IMH, 200×; (**b**) patient sample with moderate CD-163 positive structures in the epithelium and moderate to numerous CD-163 positive structures in the connective tissue (arrows), CD-163 IMH, 200×.

In the control group, the median quantity of the CD-163 positive structures in the epithelium was moderate (++), with fluctuations from none to numerous, but in the connective tissue, it was few (+), with fluctuations from rare to moderate (Figure 4a, Table 4).

The median quantity of the CD-163 positive structures in the epithelium of the patient group was rare (0/+) with fluctuations from none to few to moderate. However, the median quantity of the CD-163 positive structures in the connective tissue was a few (+) with fluctuations from none to moderate to numerous (Figure 4b, Table 5).

A comparison of the control group and patient group using the Mann–Whitney U test illustrates no statistically significant difference (U = 13.5, *p* = 0.082) in the structures of the epithelium and no statistically significant difference (U = 11.5, *p* = 0.244) in the structures of the connective tissue (Table 6).

### 3.4. IL-10

**Figure 5:**
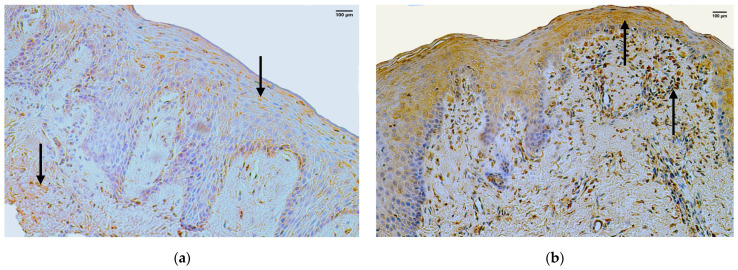
Immunohistochemistry of the IL-10 positive structures in the control and patient tissue samples. (**a**) Control sample with moderate weakly stained IL-10 positive structures in the epithelium and few IL-10 positive structures in the connective tissue (arrows), IL-10 IMH, 200×; (**b**) patient sample with numerous IL-10 positive structures in the epithelium and moderate IL-10 positive structures in the connective tissue (arrows), IL-10 IMH, 200×.

In the control group, the median quantity of the IL-10 positive structures in the epithelium was few to moderate (+/++) with fluctuations from few to moderate, but in the connective tissue, it was rare (0/+) with fluctuations from none to few (Figure 5a, Table 4).

The median quantity of IL-10 positive structures in the epithelium of the patient group was moderate (++) with fluctuations from none to abundant. The median quantity of IL-10 positive structures in the connective tissue of the patient group was few (+), with fluctuations from none to few to moderate (Figure 5b, Table 5).

A comparison of the control group and patient group using the Mann–Whitney U test illustrates no statistically significant difference (U = 18, *p* = 1.000) in the structures of the epithelium and no statistically significant difference (U = 13.5, *p* = 0.362) in the structures of the connective tissue (Table 6).

### 3.5. HBD-2

**Figure 6:**
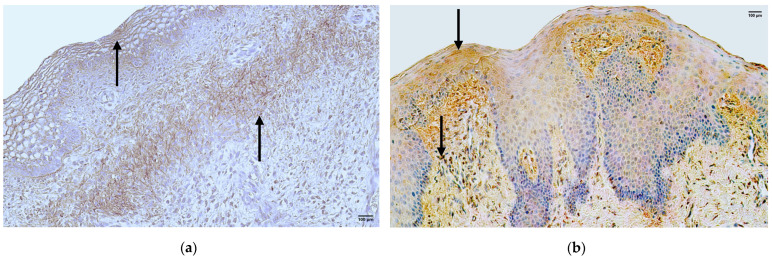
Immunohistochemistry of HBD-2 positive structures in the control and patient tissue samples. (**a**) Control sample with few to moderate HBD-2 positive structures in the epithelium and moderate HBD-2 positive structures in the connective tissue (arrows), HBD-2 IMH, 200×; (**b**) patient sample with numerous HBD-2 positive structures in the epithelium and moderate HBD-2 positive structures in the connective tissue (arrows), HBD-2 IMH, 200×.

In the control group, the median quantity of HBD-2 positive structures in the epithelium was few (+) with fluctuations from none to few to moderate, but in the connective tissue, it was rare (0/+) with fluctuations from none to moderate (Figure 6a, Table 4).

The median quantity of HBD-2 positive structures in the epithelium of the patient group was rare (0/+) with fluctuations from none to numerous. The median quantity of HBD-2 positive structures in the connective tissue of the patient group was few (+), with fluctuations from none to numerous (Figure 6b, Table 5).

A comparison of the control group and the patient group using the Mann–Whitney U test illustrates no statistically significant difference (U = 28, *p* = 0.879) in the structures of the epithelium and no statistically significant difference (U = 30.5, *p* = 0.687) in the structures of the connective tissue (Table 6).

### 3.6. HBD-3

**Figure 7:**
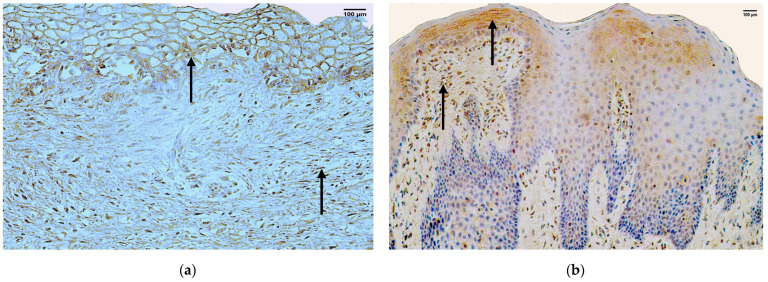
Immunohistochemistry of HBD-3 positive structures in the control and patient tissue samples. (**a**) Control sample with moderate HBD-3 positive structures in the epithelium and connective tissue (arrows), HBD-3 IMH, 200×; (**b**) patient sample with numerous HBD-3 positive structures in the epithelium and patchy moderate HBD-3 positive structures in the connective tissue (arrows), HBD-3 IMH, 200×.

In the control group, the median quantity of HBD-3 positive structures in the epithelium was few to moderate (+/++) with fluctuations from none to numerous, but in the connective tissue, it was few (+), with fluctuations from few to numerous (Figure 7a, Table 4).

The median quantity of HBD-3 positive structures in the epithelium of the patient group was rare (0/+) with fluctuations from none to numerous. The median quantity of HBD-3 positive structures in the connective tissue of the patient group was none (0), with fluctuations from none to few to moderate (Figure 7b, Table 5).

A comparison of the control group and the patient group using the Mann–Whitney U test illustrates no statistically significant difference (U = 23.5, *p* = 0.506) in the structures of the epithelium and a statistically significant difference (U = 6, *p* = 0.005) in the structures of the connective tissue (Table 6).

### 3.7. HBD-4

**Figure 8:**
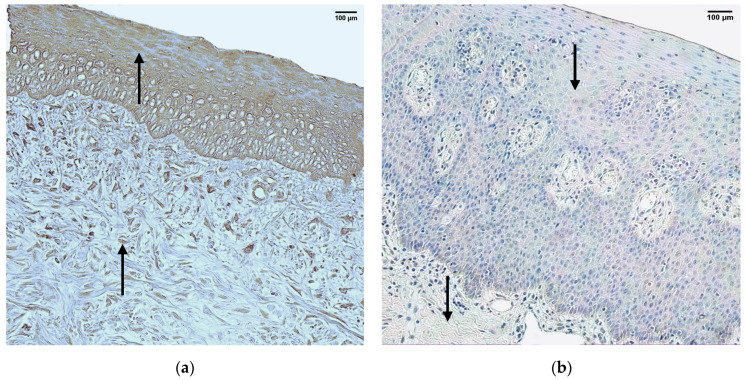
Immunohistochemistry of HBD-4 positive structures in the control and patient tissue samples. (**a**) Control sample with numerous HBD-4 positive structures in the epithelium and moderate HBD-4 positive structures in the connective tissue (arrows), HBD-4 IMH, 200×; (**b**) patient sample with no HBD-4 positive structures in the epithelium and connective tissue (arrows), HBD-4 IMH, 200×.

In the control group, the median quantity of HBD-4 positive structures in the epithelium was few (+) with fluctuations from none to numerous, but in the connective tissue, it was few (+), with fluctuations from none to moderate (Figure 8a, Table 4).

The median quantity of HBD-4 positive structures in the epithelium of the patient group was none (0), with fluctuations from none to numerous. The median quantity of HBD-4 positive structures in the connective tissue of the patient group was none (0), with fluctuations from none to rare (Figure 8b, Table 5).

A comparison of the control group and the patient group using the Mann–Whitney U test illustrates no statistically significant difference (U = 16.5, *p* = 0.16) in the structures of the epithelium and a statistically significant difference (U = 9, *p* = 0.014) in the structures of the connective tissue (Table 6).

### 3.8. Correlations in the Epithelium and the Connective Tissue of Cheilognathouranoschisis Affected Patient Group

With the use of the Spearman’s rank correlation coefficient, a strong and statistically significant correlation (r_s_ = 0.6–0.79) was obtained between the quantity of HBD-2 positive structures in the epithelium and HBD-3 positive structures in the epithelium (r_s_ = 0.697, *p* = 0.012), HBD-2 positive structures in the epithelium and HBD-4 positive structures in the epithelium (r_s_ = 0.602, *p* = 0.038), and LL-37 positive structures in the connective tissue and HBD-4 positive structures in the epithelium (r_s_ = 0.601, *p* = 0.039).

A moderate and statistically significant correlation (r_s_ = 0.4–0.59) was obtained between the quantity of HBD-2 positive structures in the connective tissue and HBD-3 positive structures in the epithelium (r_s_ = 0.575, *p* = 0.05).

The statistically significant correlations of the tissue factors in the patient group tissue samples are organized in Table 7.

## 4. Discussion

In our study, statistically significant differences between the patient group and control group tissue samples were found in HBD-3 and HBD-4 positive structures of the connective tissue (but not in the structures of the epithelium), where the patients showed a notable decrease in the occurrence of both HBD-3 and HBD-4. Thus, we speculate on the common decrease in two important local antimicrobial proteins. Interestingly, previous studies indicate that HBD-3 shares some similar anti-inflammatory functions with HBD-2 [32]. Moreover, HBD-3 notably increases the pace of tissue and wound healing with the ability to influence fibroblast activity, keratinocyte proliferation, and the release of angiogenetic growth factors [28]. On the other hand, the main antimicrobial protein HBD-2 positive structures in the patient and control groups remain statistically insignificantly different, which suggests that in orofacial cleft patients before or during milk dentition, the shared HBD-2 and HBD-3 function of anti-inflammatory actions remains unaffected. In contrast, HBD-3’s distinctive function of healing properties is severely decreased. The lack of statistically significant changes in HBD-3 immunoreactive structures in the epithelium implies that keratinocyte induction during healing processes is unaffected; however, the decrease in this tissue defense factor in the structures of connective tissues points to the fact that this irregularity directly affects the activity of fibroblasts and angiogenetic growth factor expression. This observation leads us to believe that in patients with orofacial clefts, the healing of connective tissue is suppressed, and the decrease in HBD-3 positive structures promotes the chronic state of inflammation and probably the prolonged immune response that is a distinct feature of the cleft-affected tissue. This suggestion can be explained by a study conducted by Özdemir et al., where in the process of continuous and chronic inflammation, the amount of pathogen and host organism-produced proteases increases [29]. Therefore, these proteases that act as the host organism’s protective reaction to infection and inflammation can simultaneously make HBD-3 susceptible to degradation and decrease its prevalence in the inflammation-affected tissue [29]. However, in a similar study of cleft patients of mixed dentition age conducted by Deņisova et al., the difference between the immunoreactive HBD-3 structures in the patient and control group tissue samples was not statistically significant [35]. This was because Deņisova’s study was conducted with samples obtained from older patients (during mixed dentition aged 6 to 12 years [35]), which may suggest that HBD-3 expression simultaneously and gradually increases with the maturation of the body and tissues as a protective reaction against the prolonged inflammation to successfully eliminate it and induce the healing process of damaged tissue.

HBD-4 has been previously described as a defense factor that binds to the lipopolysaccharides of bacterial cell walls and ensures antimicrobial effects combined with the decrease in TNF-α, IL-1, and TLR2 expression [33,34]. HBD-4 is also connected to the stimulated expression of IL-10, increased keratinocyte proliferation and migration, and fibroblast angiogenin synthesis [38,39]. Typically, with the presence of bacterial LPS and/or inflammatory process and tissue damage, an increased release of HBD-4 has been noted as a part of the organism’s defense mechanism [34,43,44,45,46]. Yet, in our study, HBD-4 immunoreactive structures in orofacial cleft patient tissue samples were presented in a very limited way. This suggests that the absence of HBD-4 in patients with Cheilognathouranoschisis indicates the parafunction of host tissue defense mechanisms, making the damaged tissue more susceptible to infections and the development of chronic inflammatory processes. Contrary to our results, in a study conducted by Deņisova et al., an increase in the HBD-4 positive structures was discovered in the cleft-affected tissue of patients during mixed dentition [35]. This indicates a significant difference in the expression mechanisms of HBD-4 in the tissue of cleft-affected children before and during milk dentition and in children during mixed dentition, which can be connected to the variation in teeth present. Moreover, this variation seems to affect only the cells of connective tissue because no statistically significant differences were observed in the epithelium, neither in our study nor in the study by Deņisova et al. [35]. In a study conducted by Kim et al., it was noted that the human dental pulp cells of permanent teeth secrete inflammatory factors that upregulate the expression of HBD-2 (TNF-α and IL-1) [47]. Based on these results, it can be speculated that the presence of mixed dentition and its pulp cells can also similarly upregulate the expression of HBD-4. Hence, it leads us to believe that the cells of mixed dentition and its periodontium contain some type of signaling pathways that can influence HBD-4 expression and are not present in the cells of milk dentition. In children with CLP, there possibly is a congenital defect in signaling pathways that provide normal expression of HBD-4. With the eruption of permanent teeth, different pathways are formed; thus, they can induce not only normal but also excessive expression of HBD-4. Therefore, this factor can finally carry out its functions and participate in tissue healing. It can also be speculated that the change in signaling pathways and increase in HBD-4 secretion during mixed dentition is connected to the fact that teeth eruption is a traumatic process during which the connective tissue and epithelium of the oral cavity are damaged and, therefore, more susceptible to infection and penetration of bacteria. The increase of the HBD-4 quantity possibly functions as the host organism’s defense mechanism in order to prevent the proliferation of bacteria and increase tissue healing in the site of newly erupted teeth for the sake of protection of the new structure. And, because in the cleft-affected tissue, inflammation is already present before the eruption of secondary dentition, the physiologically normal increase in HBD-4 expression over-activates as a reaction to the chronic inflammation in an effort to reduce it by stimulating keratinocyte proliferation and migration, fibroblast activity and angiogenetic factor release.

Interestingly, a presence of immunoreactive HBD-4 structures in cleft-affected tissue was observed only in tissue samples where the mother had a previous history of CLP. This could indicate a possible connection between inheritance and HBD-4 secretion regulating gene and signaling pathway expression. The results of this study, with the simultaneous decrease in both HBD-3 and HBD-4, are also consistent with a study conducted by Yanagi et al., where HBD-4 and HBD-3 showed supplementary and similar effects [43,44]. Hence, future studies can further evaluate the connection between these two factors.

A statistically notable strong positive correlation was established between the quantity of HBD-2 and HBD-3 positive structures in the epithelium. The synergy of both peptides has been previously observed in other cases of chronic inflammation, like periodontitis, chronic rhinosinusitis, and COPD [33,48,49]. It is also noted that both HBD-2 and HBD-3 share similar anti-inflammatory functions by increasing chemokine and cytokine secretion, proliferation of epithelial and connective tissue cells, like fibroblasts and keratinocytes, as well as promoting wound and damaged tissue healing [32,50]. Similar to a study by Viksne et al., the positive correlation of HBD-2 and HBD-3 in our study could be connected to changed functions of the epithelial barrier in the cleft-affected tissue for patients before or during milk dentition [49]. Moreover, a statistically significant moderate positive correlation was also identified between the quantity of HBD-3 positive structures in the epithelium and HBD-2 positive structures in the connective tissue. This would lead us to believe that in the case of orofacial cleft for children before or during milk dentition, HBD-2 expresses several functions that differ in the epithelium and the connective tissue. Based on findings of a study conducted by Umehara et al., where it was discovered that HBD-2 in the connective tissue mainly acts as an important chronic wound healing agent, it could be assumed that the presumably changed functions of the epithelial barrier with the synergetic correlation of HBD-2 and HBD-3 observed in our study, could also induce altered connective tissue cell activity with the whole purpose to promote more effective tissue healing [39].

Additionally, a statistically significant strong positive correlation was established between the quantity of HBD-2 and HBD-4 positive structures in the epithelium. HBD-2 and HBD-4 function as anti-inflammatory agents that provide the defense of the epithelium and are produced by keratinocytes in the case of upregulated expression of inflammation-modulating molecules, like TNF-α, IL-β, and INF-γ [51]. In previous studies, it has been observed that there could be a possible synergetic link between HBD-2 and HBD-4 in the role of inhibiting bacterial infection in respiratory tract tissue, as well as it has been established that the expression of these peptides can be regulated by the same factors, like retinoic acid [43,44,51]. Because HBD-4 is especially known for its ability to bind to bacterial LPS, the discovery of our study could suggest that in the case of cleft lip and palate in children aged from 6 months to 2 years, there is a presence of altered antibacterial functions of the epithelial barrier that are carried out by HBD-4 and synergistically supported by HBD-2. This correlation could also be connected to the changed quantity of HBD-4 immunoreactive structures in the connective tissue, but this needs to be examined in future research.

Furthermore, a statistically notable strong positive correlation was also observed between the quantity of LL-37 positive structures in the connective tissue and HBD-4 positive structures in the epithelium. The release of LL-37 is mainly carried out by the epithelium that coats and forms various tracts of the body, but it is also observed in neutrophils, monocytes, lymphocytes, and macrophages, which are cells that are commonly present in the connective tissue, especially in the case of inflammation [13,14]. The fact that chronic inflammation is a distinct feature of clefted tissue could be the main reason the correlation of LL-37 is only noted in connective tissue. In this tissue, LL-37 typically implements the organism’s inflammatory response with increased inflammatory and immune cell activity, characterized by increased chemotaxis, proliferation, phagocytosis, cytokine secretion, fibroblast activity, etc. [13,15]. It is also known that LL-37 protects the host body from infections not only by the exacerbation of the inflammatory response but also by directly destroying the microorganisms that have infiltrated the body with the formation of pores in the membranes and cell walls of these organisms, all as a part of innate immunity [13,15]. This activity of the factor has also been connected to the destruction of preexisting biofilms [16,17]. Therefore, keeping in mind HBD-4’s distinct feature of high affinity to bacterial LPS, it can be assumed that both of these factors function together and enhance each other’s activity in order to successfully eliminate the bacteria that has infiltrated the clefted tissue—starting with the epithelium and making its way to the connective tissue. The synergy of these factors throughout the epithelium and connective tissue could mean that they form a line of defense that protects both—epithelium and connective tissue. The co-action of LL-37 and HBD-4 has also been identified in previous studies conducted by Deņisova et al. and Niyonsaba et al., where the shared function of IL-18 and IL-31 secretion and the mutual regulation through TNF-α was noted [34,35,38,52,53].

Interestingly, in our study, no statistically significant changes or correlations were observed in the immunohistochemical analysis of IL-10. In previous similar studies, the link between HBDs and IL-10 expression has been noted, with the mention that in the case of inflammation, HBDs usually upregulate the expression and secretion of IL-10 [35,38,54,55]. However, in our study, no such observation was made; therefore, it leads us to believe that in cleft-affected tissue for children aged from 6 months to 2 years, there is a factor that suppresses the typically observed upregulated expression of IL-10. It is possible that this suppression is carried out by LL-37, which has shown a positive correlation with HBD-4 in this study. This theory is supported by previous studies, which also concluded that LL-37 has a proinflammatory effect and reduces the secretion of anti-inflammatory cytokine IL-10 [19,56,57].

In addition, it is also noteworthy that not only M2 macrophages but also other similar CD-163 immunoreactive substances produced by M2 macrophages were visible in the epithelium. This leads us to believe that in CLP children aged from 6 months to 2 years, there is a presence of immunohistochemical cross-reactivity that needs to be examined further in future studies.

The suspected link between the examined tissue defense factors and inflammatory processes of the orofacial region suggests that the deficiency of these factors promotes the development of inflammation. Therefore, they could be potentially used as a therapeutic chronic inflammation treatment option that would aid the epithelium and connective tissue of the oral cavity in faster and more effective healing in the case of prolonged inflammation and/or after surgical treatment of the defect [23,30,31,58,59,60,61]. The use of these factors could decrease healing time, scar tissue formation, development of tissue healing complications and significantly improve the outcome and overall recovery process of the patient [23].

Our study is a part of 20 years of research projects, and we have observed a novel difference in distribution and fluctuations of defense factors in cleft-affected palate tissue in children before and during milk dentition. Previous studies show that early childhood demonstrates the different distribution of the same defense factors (HBD-2, HBD-4, IL-10 [35]); therefore, we can conclude that defense factor expression changes with childhood development and maturation of the tissue.

A notable limitation in our study could be that some patient group samples lacked epithelium because the acquisition of tissue samples during veloplastic surgery is challenging. The presence of orofacial cleft has already created a deficiency of tissue material; therefore, every single piece of tissue available is needed to complete a successful surgery that renews the damaged tissue as much as possible. Hence, not much is left to obtain for scientific research purposes. A partial limitation in our study could be that there is no correlation between the local defense factor quantitative changes and clinical signs and symptoms of the patients, for example, biomechanical defects, functional impediments etc. This marks a possible future research plan to connect immunohistochemical indices with clinical manifestations and indications for possible treatment with various biomaterials [62].

Some other methods, like ELISA and in situ hybridization, can clarify the local tissue defense factor concentration and the gene assay in the cleft-affected tissue.

In summary, a statistically notable decrease in defense factor expression was noted when comparing the HBD-3 and HBD-4 positive structures in the connective tissue. This could suggest a possible promotion of chronic inflammatory processes. The deficiency of HBD-4 could be associated with signaling pathways that are specific to dental pulp cells. Furthermore, several significant strong and moderate mutual correlations were also observed between HBD-2, HBD-3, HBD-4, and LL-37 IHC-positive structures of both the epithelium and connective tissue. This indicates noteworthy changes in the epithelial barrier, amplification of healing processes, and an increase in antibacterial defense mechanisms. In addition, no notable changes were discovered in IL-10 expression; therefore, a possible suppression of the factor could be present in patients with CLP. Interestingly, we also noted that the presence of M2 macrophage produced similar CD-163 immunoreactive substances.

## 5. Conclusions

A decreased amount of HBD-3 in the connective tissue implies downregulated fibroblast and angiogenetic growth factor expression, promoting a chronic state of inflammation and prolonged immune response in cleft-affected tissue.

Almost the absence of HBD-4 in connective tissue indicates the parafunction of host tissue defense mechanisms that increase susceptibility to infections of the cleft-affected palate. The deviance in the HBD-4 levels in children before and during milk dentition age compared to the mixed dentition age could be connected to tissue defense factors signaling pathways that are specific to permanent dentition.

A strong positive correlation between HBD-2 and HBD-4 in the epithelium suggests the mutually and synergistically supported alteration of antibacterial functions of the epithelial barrier in patients with CLP.

The synergy of LL-37 and HBD-4 indicates the protection of epithelium and connective tissue by enhancing each other’s ability to eliminate bacteria.

Future research could include gene detection, defense factor concentration detection, and correlation with clinical data in the longitudinal aspect as a final outcome after all plastic surgeries have been performed on the cleft-affected children.

## Figures and Tables

**Table 1:** Information about the patients.

Patient Number	Age (Months)	Sex	Diagnosis	Surgery	Remarks
237	8	M	Cheilognathouranoschisis sinistra	Veloplastic	Mother with CLP
285	8	F	Cheilognathouranoschisis dextra	Veloplastic	
319	8	F	Cheilognathouranoschisis sinistra	Veloplastic	
335/1	8	F	Cheilognathouranoschisis bilateralis	Veloplastic	
335/2	8	F	Cheilognathouranoschisis bilateralis	Veloplastic	
261	9	M	Cheilognathouranoschisis bilateralis	Veloplastic	
276	9	M	Cheilognathouranoschisis bilateralis	Veloplastic	
326	9	F	Cheilognathouranoschisis sinistra	Veloplastic	
332	9	M	Cheilognathouranoschisis dextra	Veloplastic	
366	9	M	Cheilognathouranoschisis sinistra	Veloplastic	
233	10	M	Cheilognathouranoschisis dextra	Veloplastic	
298	11	F	Cheilognathouranoschisis sinistra	Veloplastic	Mother smoked during pregnancy
17	12	F	Cheilognathouranoschisis dextra	Veloplastic	
362	12	F	Cheilognathouranoschisis dextra	Veloplastic	Paracetamol during pregnancy

Abbreviations: M—male; F—female; CLP—cleft lip and palate.

**Table 2:** Information about the control group.

Control Number	Age	Sex	Cause of Death
2b	Newborn	M	Asphyxia by the umbilical cord
3b	Newborn	F	Asphyxia by the umbilical cord
4b	24 weeks	F	Abortion due to the maternal health status
5b	Newborn	F	Sudden death syndrome
6b	Newborn	F	Sudden death syndrome

Abbreviations: M—male; F—female.

**Table 3:** Identifiers used in the semi-quantitative evaluation of tissue defense factors [40,41].

Identifier Used	Explanation
0	No positive structures in the visual field (0%)
0/+	Rare occurrence of positive structures in the visual field (12.5%)
+	Few positive structures in the visual field (25%)
+/++	Few to moderate numbers of positive structures in the visual field (37.5%)
++	Moderate number of positive structures in the visual field (50%)
++/+++	Moderate to numerous positive structures in the visual field (62.5%)
+++	Numerous positive structures in the visual field (75%)
+++/++++	Numerous to abundant positive structures in the visual field (87.5%)
++++	Abundance of positive structures in the visual field (100%)

**Table 4:** Semi-quantitative evaluation of defense factors in the epithelium and connective tissue for the control group.

Sample Number	LL-37		CD-163		IL-10		HBD-2		HBD-3		HBD-4	
	E	CT	E	CT	E	CT	E	CT	E	CT	E	CT
2b	0	++	0	+	U	U	0	++	0	+++	0	++
3b	+/++	++	+	++	++	0	+/++	0	+++	++	+	+
4b	+++	++	U	U	+	+	+/++	+	0	+	0	+
5b	++/+++	++	U	U	++	+	0	0/+	++	+	++	0
6b	+/++	++	++	++	U	U	+	0	+/++	+	+++	++
Median	+/++	++	+	++	++	+	+	0/+	+/++	+	+	+

Abbreviations: IL-10—interleukin 10; HBD-2—human beta-defensin 2; HBD-3—human beta-defensin 3; HBD-4—human beta-defensin 4; E—epithelium; CT—connective tissue; U—unable to determine.

**Table 5:** Semi-quantitative evaluation of defense factors in the epithelium and connective tissue for the patient group with Cheilognathouranoschisis.

Sample Number	LL-37		CD-163		IL-10		HBD-2		HBD-3		HBD-4	
	E	CT	E	CT	E	CT	E	CT	E	CT	E	CT
237	++++	+++	+/++	+++	+++	+	+++	++	+++	0	+++	0
285	++	+++	0	+	0	0/+	0	0	0	0	0	0
319	+/++	+++	+/++	+	++	+	+/++	0/+	0/+	0	0/+	0
335/1	++/+++	++	0	0	0/+	0	+/++	0	0/+	0	0	0
335/2	+/++	0	0/+	0	+++	+	0	0/+	0	0	0	+
261	++	++	+/++	++/+++	+/++	0/+	0	0	0	0/+	0	0
276	+	0	0	++/+++	++	++	0	++	++	0	0	0
326	+++	+/++	+	0	+++	0/+	++/+++	+/++	++	0	0	0
332	0	++	+	0	+/++	++	0/+	+	+	+	0	0
366	0	0	0	0/+	0	0/+	0	+++	0	0	0	0
233	N	++	N	+/++	N	++	N	+/++	N	+/++	N	0
298	+/++	+/++	0/+	+	++++	+/++	0	0	0	0	0	0
17	N	0	N	0	N	++	N	+/++	N	+	N	0/+
362	0	0	0	0/+	0	+/++	0/+	0	0	0	0	0
Median	+/++	++	0/+	+	++	+	0/+	+	0/+	0	0	0

Abbreviations: IL-10—interleukin 10; HBD-2—human beta-defensin 2; HBD-3—human beta-defensin 3; HBD-4—human beta-defensin 4; E—epithelium; CT—connective tissue; N—no epithelium.

**Table 6:** Comparison of the patient and control group median values for semi-quantitively evaluated defense factors in the epithelium and connective tissue.

9	LL-37		CD-163		IL-10		HBD-2		HBD-3		HBD-4	
	E	CT	E	CT	E	CT	E	CT	E	CT	E	CT
Patient group	+/++	++	0/+	+	++	+	0/+	+	0/+	0	0	0
Control group	+/++	++	++	+	++	+	+	0/+	+/++	+	+	+
U-test value	27.5	25.0	13.5	11.5	18.0	13.5	28.0	30.5	23.5	6.0	16.5	9.0
*p*-value	0.799	0.391	0.082	0.244	1.000	0.362	0.879	0.687	0.506	0.005	0.160	0.014

Abbreviations: IL-10—interleukin 10; HBD-2—human beta-defensin 2; HBD-3—human beta-defensin 3; HBD-4—human beta-defensin 4; E—epithelium; CT—connective tissue; U-test value—Mann–Whitney U test value.

**Table 7:** Statistically notable Spearman’s rank correlation coefficient comparison analysis between tissue defense factors in the patient group.

Strength of Correlation	Correlations between Tissue Defense Factors in Patient Group	r_s_	*p*-Value
Strong association (0.6–0.79)	HBD-2 in epithelium and HBD-3 in the epithelium	0.697	0.012
	HBD-2 in epithelium and HBD-4 in the epithelium	0.602	0.038
	LL-37 in connective tissue and HBD-4 in the epithelium	0.601	0.039
Moderate association (0.4–0.59)	HBD-2 in connective tissue and HBD-3 in the epithelium	0.575	0.050

Abbreviations: HBD-2—human beta-defensin 2; HBD-3—human beta-defensin 3; HBD-4—human beta-defensin 4; r_s_—Spearman’s rho value.

## Data Availability

All datasets used in the present study are available in the Section 3.

## References

[1] Nasreddine G., El Hajj J., Ghassibe-Sabbagh M. (2021). Orofacial clefts embryology, classification, epidemiology, and genetics. Mutat. Res. Rev. Mutat. Res..

[2] Pilmane M., Jain N., Jain S., Akota I., Kroiča J. (2021). Quantification of Cytokines in Lip Tissue from Infants Affected by Congenital Cleft Lip and Palate. Children.

[3] Kempa I., Ambrozaitytė L., Stavusis J., Akota I., Barkane B., Krumina A., Matulevičienė A., Utkus A., Kučinskas V., Lace B. (2014). Association of BMP4 polymorphisms with non-syndromic cleft lip with or without cleft palate and isolated cleft palate in Latvian and Lithuanian populations. Stomatologija.

[4] Vaivads M., Akota I., Pilmane M. (2023). Characterization of SHH, SOX3, WNT3A and WNT9B Proteins in Human Non-Syndromic Cleft Lip and Palate Tissue. Dent. J..

[5] Worley M.L., Patel K.G., Kilpatrick L.A. (2018). Cleft Lip and Palate. Clin. Perinatol..

[6] Vieira A.R., Pliss L., Pelnena I., Krumina A., Baumanis V., Lace B. (2011). Mitochondrial DNA origins of the Latvian clefting population. Mitochondrion.

[7] Lithovius R.H., Ylikontiola L.P., Harila V., Sándor G.K. (2014). A descriptive epidemiology study of cleft lip and palate in Northern Finland. Acta Odontol. Scand..

[8] Goida J., Pilmane M. (2022). The Evaluation of FGFR1, FGFR2 and FOXO1 in Orofacial Cleft Tissue. Children.

[9] Jankovska I., Pilmane M., Akota I. (2017). Expression of gene proteins, interleukins and β-defensin in cleft-affected tissue. Stomatologija.

[10] Salari N., Darvishi N., Heydari M., Bokaee S., Darvishi F., Mohammadi M. (2022). Global prevalence of cleft palate, cleft lip and cleft palate and lip: A comprehensive systematic review and meta-analysis. J. Stomatol. Oral Maxillofac. Surg..

[11] Zhou F., Su Z., Li Q., Wang R., Liao Y., Zhang M., Li J. (2022). Characterization of Bacterial Differences Induced by Cleft-Palate-Related Spatial Heterogeneity. Pathogens.

[12] Costa B., Lima J.E., Gomide M.R., Rosa O.P. (2003). Clinical and microbiological evaluation of the periodontal status of children with unilateral complete cleft lip and palate. Cleft Palate Craniofacial J..

[13] Nakamichi Y., Horibe K., Takahashi N., Udagawa N. (2014). Roles of cathelicidins in inflammation and bone loss. Odontology.

[14] Khurshid Z., Naseem M., Yahya I.A.F., Mali M., Sannam Khan R., Sahibzada H.A., Zafar M.S., Faraz Moin S., Khan E. (2017). Significance and Diagnostic Role of Antimicrobial Cathelicidins (LL-37) Peptides in Oral Health. Biomolecules.

[15] Chinipardaz Z., Zhong J.M., Yang S. (2022). Regulation of LL-37 in Bone and Periodontium Regeneration. Life.

[16] Chen X., Zou X., Qi G., Tang Y., Guo Y., Si J., Liang L. (2018). Roles and Mechanisms of Human Cathelicidin LL-37 in Cancer. Cell. Physiol. Biochem..

[17] Chen X., Ji S., Si J., Zhang X., Wang X., Guo Y., Zou X. (2020). Human cathelicidin antimicrobial peptide suppresses proliferation, migration and invasion of oral carcinoma HSC-3 cells via a novel mechanism involving caspase-3 mediated apoptosis. Mol. Med. Rep..

[18] Greer A., Zenobia C., Darveau R.P. (2013). Defensins and LL-37: A review of function in the gingival epithelium. Periodontol. 2000.

[19] Moreno-Angarita A., Aragón C.C., Tobón G.J. (2020). Cathelicidin LL-37: A new important molecule in the pathophysiology of systemic lupus erythematosus. J. Transl. Autoimmun..

[20] Ferrisse T.M., de Oliveira A.B., Palaçon M.P., Silva E.V., Massucato E.M.S., de Almeida L.Y., Léon J.E., Bufalino A. (2021). The role of CD68+ and CD163+ macrophages in immunopathogenesis of oral lichen planus and oral lichenoid lesions. Immunobiology.

[21] Sun X., Gao J., Meng X., Lu X., Zhang L., Chen R. (2021). Polarized Macrophages in Periodontitis: Characteristics, Function, and Molecular Signaling. Front. Immunol..

[22] Lyu J., Bian T., Chen B., Cui D., Li L., Gong L., Yan F. (2017). β-defensin 3 modulates macrophage activation and orientation during acute inflammatory response to Porphyromonas gingivalis lipopolysaccharide. Cytokine.

[23] Nakao Y., Fukuda T., Zhang Q., Sanui T., Shinjo T., Kou X., Chen C., Liu D., Watanabe Y., Hayashi C. (2021). Exosomes from TNF-α-treated human gingiva-derived MSCs enhance M2 macrophage polarization and inhibit periodontal bone loss. Acta Biomater..

[24] Etzerodt A., Moestrup S.K. (2013). CD163 and inflammation: Biological, diagnostic, and therapeutic aspects. Antioxid. Redox Signal.

[25] Ouyang W., O’Garra A. (2019). IL-10 Family Cytokines IL-10 and IL-22: From Basic Science to Clinical Translation. Immunity.

[26] Rutz S., Ouyang W. (2016). Regulation of Interleukin-10 Expression. Adv. Exp. Med. Biol..

[27] Saraiva M., Vieira P., O’Garra A. (2020). Biology and therapeutic potential of interleukin-10. J. Exp. Med..

[28] Takahashi M., Umehara Y., Yue H., Trujillo-Paez J.V., Peng G., Nguyen H.L.T., Ikutama R., Okumura K., Ogawa H., Ikeda S. (2021). The Antimicrobial Peptide Human β-Defensin-3 Accelerates Wound Healing by Promoting Angiogenesis, Cell Migration, and Proliferation Through the FGFR/JAK2/STAT3 Signaling Pathway. Front. Immunol..

[29] Özdemir M., Caglayan F., Bikker F.J., Pussinen P., Könönen E., Yamalik N., Gürsoy M., Fteita D., Nazmi K., Güncü G.N. (2020). Gingival tissue human beta-defensin levels in relation to infection and inflammation. J. Clin. Periodontol..

[30] Yilmaz D., Topcu A.O., Akcay E.U., Altındis M., Gursoy U.K. (2020). Salivary human beta-defensins and cathelicidin levels in relation to periodontitis and type 2 diabetes mellitus. Acta Odontol. Scand..

[31] Cieślik M., Bagińska N., Górski A., Jończyk-Matysiak E. (2021). Human β-Defensin 2 and Its Postulated Role in Modulation of the Immune Response. Cells.

[32] Judge C.J., Reyes-Aviles E., Conry S.J., Sieg S.S., Feng Z., Weinberg A., Anthony D.D. (2015). HBD-3 induces NK cell activation, IFN-γ secretion and mDC dependent cytolytic function. Cell Immunol..

[33] Vitenberga Z., Pilmane M., Babjoniševa A. (2019). An Insight into COPD Morphopathogenesis: Chronic Inflammation, Remodeling, and Antimicrobial Defense. Medicina.

[34] Zhai Y., Wang Y., Rao N., Li J., Li X., Fang T., Zhao Y., Ge L. (2019). Activation and Biological Properties of Human β Defensin 4 in Stem Cells Derived From Human Exfoliated Deciduous Teeth. Front. Physiol..

[35] Deņisova A., Pilmane M., Kažoka D. (2023). Antimicrobial Peptides and Interleukins in Cleft Soft Palate. Children.

[36] Scheid R.C., Weiss G. (2012). Woelfel’s Dental Anatomy.

[37] Suvarna S.K., Layton C., Bancroft J.D. (2019). Bancroft’s Theory and Practice of Histological Techniques.

[38] Niyonsaba F., Ushio H., Nakano N., Ng W., Sayama K., Hashimoto K., Nagaoka I., Okumura K., Ogawa H. (2007). Antimicrobial peptides human beta-defensins stimulate epidermal keratinocyte migration, proliferation and production of proinflammatory cytokines and chemokines. J. Investig. Dermatol..

[39] Umehara Y., Takahashi M., Yue H., Trujillo-Paez J.V., Peng G., Nguyen H.L.T., Okumura K., Ogawa H., Niyonsaba F. (2022). The Antimicrobial Peptides Human β-Defensins Induce the Secretion of Angiogenin in Human Dermal Fibroblasts. Int. J. Mol. Sci..

[40] Vaivads M., Akota I., Pilmane M. (2021). Cleft Candidate Genes and Their Products in Human Unilateral Cleft Lip Tissue. Diseases.

[41] Vitenberga Z., Pilmane M., Babjoniševa A. (2019). The evaluation of inflammatory, anti-inflammatory and regulatory factors contributing to the pathogenesis of COPD in airways. Pathol. Res. Pract..

[42] Barton B., Peat J. (2014). Medical Statistics: A Guide to SPSS, Data Analysis and Critical Appraisal.

[43] Yanagi S., Ashitani J., Ishimoto H., Date Y., Mukae H., Chino N., Nakazato M. (2005). Isolation of human beta-defensin-4 in lung tissue and its increase in lower respiratory tract infection. Respir. Res..

[44] Yanagi S., Ashitani J., Imai K., Kyoraku Y., Sano A., Matsumoto N., Nakazato M. (2007). Significance of human beta-defensins in the epithelial lining fluid of patients with chronic lower respiratory tract infections. Clin. Microbiol. Infect..

[45] Paris S., Wolgin M., Kielbassa A.M., Pries A., Zakrzewicz A. (2009). Gene expression of human beta-defensins in healthy and inflamed human dental pulps. J. Endod..

[46] Noronha S.A., Noronha S.M., Lanziani L.E., Ipolito M.Z., Ferreira L.M., Gragnani A. (2014). Human beta defensin-4 and keratinocyte growth factor gene expression in cultured keratinocyte and fibroblasts of burned patients. Acta Cir. Bras..

[47] Kim Y.S., Min K.S., Lee S.I., Shin S.J., Shin K.S., Kim E.C. (2010). Effect of proinflammatory cytokines on the expression and regulation of human beta-defensin 2 in human dental pulp cells. J. Endod..

[48] Zhang C., Han Y., Miao L., Yue Z., Xu M., Liu K., Hou J. (2023). Human β-defensins are correlated with the immune infiltration and regulated by vitamin D(3) in periodontitis. J. Periodontal Res..

[49] Viksne R.J., Sumeraga G., Pilmane M. (2023). Antimicrobial and Defense Proteins in Chronic Rhinosinusitis with Nasal Polyps. Medicina.

[50] Fusco A., Savio V., Donniacuo M., Perfetto B., Donnarumma G. (2021). Antimicrobial Peptides Human Beta-Defensin-2 and -3 Protect the Gut During Candida albicans Infections Enhancing the Intestinal Barrier Integrity: In Vitro Study. Front. Cell Infect. Microbiol..

[51] Harder J., Meyer-Hoffert U., Wehkamp K., Schwichtenberg L., Schröder J.M. (2004). Differential gene induction of human beta-defensins (hBD-1, -2, -3, and -4) in keratinocytes is inhibited by retinoic acid. J. Investig. Dermatol..

[52] Niyonsaba F., Ushio H., Nagaoka I., Okumura K., Ogawa H. (2005). The human beta-defensins (-1, -2, -3, -4) and cathelicidin LL-37 induce IL-18 secretion through p38 and ERK MAPK activation in primary human keratinocytes. J. Immunol..

[53] Huang L.C., Petkova T.D., Reins R.Y., Proske R.J., McDermott A.M. (2006). Multifunctional roles of human cathelicidin (LL-37) at the ocular surface. Investig. Ophthalmol. Vis. Sci..

[54] Cui D., Lyu J., Li H., Lei L., Bian T., Li L., Yan F. (2017). Human β-defensin 3 inhibits periodontitis development by suppressing inflammatory responses in macrophages. Mol. Immunol..

[55] Kanda N., Kamata M., Tada Y., Ishikawa T., Sato S., Watanabe S. (2011). Human β-defensin-2 enhances IFN-γ and IL-10 production and suppresses IL-17 production in T cells. J. Leukoc. Biol..

[56] van der Does A.M., Beekhuizen H., Ravensbergen B., Vos T., Ottenhoff T.H., van Dissel J.T., Drijfhout J.W., Hiemstra P.S., Nibbering P.H. (2010). LL-37 directs macrophage differentiation toward macrophages with a proinflammatory signature. J. Immunol..

[57] Soldati K.R., Toledo F.A., Aquino S.G., Rossa C., Deng D., Zandim-Barcelos D.L. (2021). Smoking reduces cathelicidin LL-37 and human neutrophil peptide 1-3 levels in the gingival crevicular fluid of patients with periodontitis. J. Periodontol..

[58] Yılmaz D., Güncü G.N., Könönen E., Barış E., Çağlayan F., Gursoy U.K. (2015). Overexpressions of hBD-2, hBD-3, and hCAP18/LL-37 in Gingiva of Diabetics with Periodontitis. Immunobiology.

[59] Ballestas S.A., Turner T.C., Kamalakar A., Stephenson Y.C., Willett N.J., Goudy S.L., Botchwey E.A. (2019). Improving hard palate wound healing using immune modulatory autotherapies. Acta Biomater..

[60] Wang G., Narayana J.L., Mishra B., Zhang Y., Wang F., Wang C., Zarena D., Lushnikova T., Wang X. (2019). Design of Antimicrobial Peptides: Progress Made with Human Cathelicidin LL-37. Adv. Exp. Med. Biol..

[61] Ridyard K.E., Overhage J. (2021). The Potential of Human Peptide LL-37 as an Antimicrobial and Anti-Biofilm Agent. Antibiotics.

[62] Hareharen K., Kumar P., Panneerselvam T., Babu D., Sriraman N. (2023). Investigating the effect of laser shock peening on the wear behaviour of selective laser melted 316L stainless steel. Opt. Laser Technol..

